# Exposure to Copper Compromises the Maturational Competency of Porcine Oocytes by Impairing Mitochondrial Function

**DOI:** 10.3389/fcell.2021.678665

**Published:** 2021-06-04

**Authors:** Jingyue Chen, Zhaokang Cui, Yawei Qiu, Xingxing Zhang, Fang Chen, Huili Wang, Bo Xiong, Yilong Miao, Qian Gao

**Affiliations:** ^1^College of Animal Science and Technology, Nanjing Agricultural University, Nanjing, China; ^2^College of Veterinary Medicine, Nanjing Agricultural University, Nanjing, China; ^3^Institute of Animal Science, Jiangsu Academy of Agricultural Sciences, Nanjing, China

**Keywords:** Cu, ROS, oocyte quality, cytoskeleton, mitochondrial function

## Abstract

Copper (Cu) is an essential trace element for animals, and also an important nutritional component for the normal physiology and metabolism of animal reproductive systems. An excess or lack of Cu will directly or indirectly affect animal reproductive activities. However, the effect of Cu, in particular excessive Cu, on the reproductive performance of sows has not been studied. Here, we report that excessive Cu had negative effects on oocyte maturation and organelle functions. We showed that Cu exposure perturbed porcine oocyte meiotic maturation and impaired spindle/chromosome structure, resulting in a defective spindle assembly, as well as the abnormal distribution of actin dynamics and cortical granules. In addition, single-cell transcriptome analysis identified the target effectors of Cu actions in porcine oocytes, further demonstrating that Cu exposure affects the mitochondrial distribution and function, leading to the high levels of reactive oxygen species, DNA damage, and early apoptosis of porcine oocytes. These findings demonstrate that Cu exposure causes abnormalities in the mitochondrial distribution and function, resulting in the increased oxidative stress and levels of reactive oxygen species, DNA damage, and apoptosis, ultimately leading to a decreased porcine oocyte quality.

## Introduction

Copper (Cu) is an essential trace element that plays an important role in a normal growth, development, metabolism, and reproduction. However, excessive Cu content may cause adverse effects within organisms (Yin et al., 2018). Excessive heavy metal levels pose a serious problem for animal feed safety in China. Enrichment of heavy metals in soil of forage crops or the addition of heavy metals may lead to excessive levels in animal feed (Hejna et al., 2018). The unsuitable use of Cu, a heavy metal, has adversely affected livestock production and, more importantly, excessive addition of dietary Cu to livestock has led to serious environmental problems via its excretion in feces (Sutton and Richert, 2004). Cu pollution in the environment could lead to Cu poisoning in animals, and sheep are very sensitive to Cu. Cu poisoning in sheep can cause renal degeneration, hemolysis, and central nervous system diseases (Dewes, 1996; Grace, 2006). What is more, Cu pollution of environmental contaminants may influence a behavioral problem in children, and child Cu exposure was associated with higher internalizing symptoms (Maitre et al., 2021).

Pigs are non-ruminants and generally do not suffer from Cu deficiency. It is rarely necessary to add additional Cu to the diet of pigs to maintain the minimum Cu requirements (Hill and Shannon, 2019). In 1948, researchers found that pigs that licked Cu pipes grew rapidly, and it was proposed that Cu at pharmacological concentrations of 125–250 ppm could promote the growth of pigs. The concentration of Cu added to the diets of weaned piglets was often higher than the demand of normal animals for Cu. Cu added to a diet at 242 ppm was considered the maximum dose to stimulate the growth of pigs (Cromwell et al., 1989), and 500 ppm was toxic (Suttle and Mills, 1966). Many other studies have shown that a diet high in Cu can improve the growth performance of weaned piglets, as well as increase the final body weight and hot carcass weight of pigs (Wang et al., 2016; Coble et al., 2017, 2018). Although feeds with a high Cu content can stimulate the growth of microorganisms and lipase secretion in pigs, promote more eating, accelerate growth, and improve feed utilization (Hill et al., 2000; Hasman et al., 2006; Debski, 2016), heavy metals absorbed by livestock in large doses can cause severe gastrointestinal symptoms and damage to the liver, kidneys, and central nervous system (Wilkinson et al., 2003).

There are not many studies on the actions of Cu in domestic animal reproduction, with most focusing on the *in vitro* culture of bovine oocytes and ovine sperm development (Picco et al., 2012; Rosa et al., 2016). The effect of Cu, in particular excessive Cu, on the reproductive performance of boars and sows has not been studied. In this paper, we performed a systematic study into the effects of Cu pollution on the quality and reproductive performance of sow oocytes, and identified potential molecular markers to evaluate oocyte quality and targets of heavy metal pollution, and develop strategies and solutions to improve the reproductive health and breeding technology of pigs. This study used the single-cell transcriptome analysis to explore the mechanism of Cu actions on porcine oocyte quality. The current findings expand our understanding on the effect of environmental pollutants on female gamete development.

## Materials and Methods

### Antibodies

Mouse monoclonal α-tubulin-FITC (fluorescein isothiocyanate), phalloidin-TRITC (actin), lens culinaris agglu-tinin (LCA)-FITC, and PI (Propidium Iodide) were obtained from Sigma-Aldrich (St Louis, MO, United States). Alexa Fluor 488 goat anti-rabbit and 594 goat anti-mouse antibodies were from Invitrogen (Carlsbad, CA, United States). Rabbit polyclonal human γH2AX antibodies were obtained from Cell Signaling Technology (Danvers, MA, United States).

### Collection of Porcine Oocytes

Porcine ovaries were taken from a local slaughterhouse and transported to the laboratory within two hours of slaughter in saline containing streptomycin sulfate and penicillin G. Cumulus-oocyte complexes (COCs) were aspirated from the follicles with a disposable syringe. COCs with dense cumulus cells were selected for the subsequent *in vitro* maturation (IVM). TCM-199 was used as the maturation medium (Thermo Fisher Scientific, United States; Cat# 11150059) and the medium was supplemented with 10% porcine follicular fluid, 10 ng/mL of EGF, 5 μg/mL of insulin, 0.2 mM pyruvate, 25 μg/mL of kanamycin, 0.6 mM cysteine, and 10 IU/mL of each hCG and eCG. Twenty germinal vesicle (GV) COCs were placed in a drop of 100 μL maturation medium as mentioned above, covered with mineral oil, and cultured at 38.5°C, 5% CO_2_ for 26–28 h to metaphase I stage, and for 42–44 h to metaphase II stage.

### Cu Treatment

Copper sulfate standard solution(Macklin;Cat# 7758-98-7)was added to the maturation medium until it reached a volume of 500 μg/mL, which was further diluted to the working concentration of 15, 25, 50, or 100 μg/mL, respectively, with maturation medium. We thus chose 25 μg/ml for our subsequent study because this concentration not only has adverse effects on the oocyte maturation but also allowed us to collect matured oocytes for other experiments.

### Fluorescence Staining and Confocal Microscopy

Denuded oocytes (DOs) were treated with the fixation solution (4% paraformaldehyde in PBS) for 30 min. Then, these DOs were incubated in the permeabilization solution (1% Triton X-100 in PBS) for one hour, and after incubating in the blocking solution (1% BSA-supplemented PBS) at room temperature (RT) for one hour, they were incubation with lens culinaris agglu-tinin (LCA)-FITC, α-tubulin-FITC antibody (1:200), γ-H2AX antibody (1:100), or phalloidin-TRITC (1:100) at 4°C for overnight. These oocytes were washed in the PBST, and incubated with the corresponding secondary antibodies for one hour. Next, they were counterstained with propidium iodide (PI) or Hoechst 33342 (10 μg/ml) at RT for 10 min. In addition, the oocytes were stained for 30 min at 38.5°C in the MitoTracker Red CMXRos (500 nM) for mitochondrion staining, in the MitoProbe JC-1 (2 μM) for mitochondrial membrane potential assessment, and in the dichlorofluorescein diacetate (DCFHDA, 10 μM) for ROS staining, and with Annexin-V-FITC (1:10; Beyotime, Huangzhou, China) for apoptosis assessment. Lastly, oocytes were mounted on the glass slides and imaged under a confocal microscope (LSM 700 META, Zeiss, Germany).

### RNA Extraction and Quantitative Real-Time PCR

Fifty oocytes were collected and the total RNA was extracted using the RNeasy Mini Kit (Qiagen, United States), which was then reversed to the cDNA using PrimeScript RT Master Mix (Takara, Japan). Real-time quantitative PCR was performed using the SYBR Green PCR Master Mix (ThermoFisher Scientific). All data were normalized by GAPDH, and the comparative CT method was used to quantify the fold changes. The list of primers used was as follows (Miao et al., 2021):

*Atp5pf* (F: TCAGTCTGCGGTCTCGG/R: CTCAGGGCCAG CATCAA);

*Ndufb3* (F: TGGAAGATAGAAGGGACA/R: GCAAAGCCA CCAGAGTA);

*Ndufab1* (F: CCGTGTCCTTTACGTCTTG/R: TGGGCAC ATTAACTTCTCC);

*Ndufa13* (F: GATGAAGTGGAACCGTGAG/R: TCCGCAG CCCATAGAGC);

*GSR* (F: ACAGTGGGACTCACAGAAGA/R: AGGTAGG ATGAATGGCAAC);

*GPX1* (F: CCAAGTTTATCACCTGGTCTCC/R: AGGCACT GCTAGGCTCCTG);

*GPX4* (F: TGTGGTTTACGGATTCTGG/R: CCTTGGGC TGGACTTTCA);

*SOD1* (F: GGTCCTCACTTCAATCCTG/R: CTTCATT TCCACCTCTGC);

*SOD2* (F: TATCCGTCGGCGTCCAAG/R: GCGGCGTAT CGCTCAGTT); and

*GAPDH* (F: TGGGCTACACTGAGGACC/R: TACCAGGA AATGAGCTTGA).

### Single-Cell RNA Library Construction and Transcriptome Sequencing

Transcriptome analysis of mature oocytes was performed with the single cell RNA-Seq protocol. In short, three sets of samples (three oocytes per sample) were collected for each group and placed in the lysis buffer. The single cell collection solution contains the RNase inhibitors and cell lysis components. The nucleic acid sequence was used for reverse transcription with oligo dT to form the 1st cDNA. The 1st cDNA was amplified by PCR, nucleic acid was enriched, and the library was constructed after purification of the amplified products, including DNA fragmentation, terminal repair, adding “A” and joint, PCR amplification, and library quality control. The constructed library was sequenced on the Illumina platform. The sequencing strategy was PE150. The raw down sequence (Raw Reads) obtained by Hiseq sequencing were completed by a process of removing the low-quality sequence and connector pollution. Then, high-quality sequences (clean reads) were obtained, and all subsequent analysis was based on the clean reads (Miao et al., 2020). The sequencing data was filtered with SOAPnuke (v1.5.2) (Li et al., 2008) by (1) Removing reads containing sequencing adapter; (2) Removing reads whose low-quality base ratio (base quality less than or equal to 5) is more than 20%; and (3) Removing reads whose unknown base (“N” base) ratio is more than 5%. Afterward, the clean reads were obtained and stored in the FASTQ format. The clean reads were mapped to the reference genome using HISAT2 (v2.0.4) (Kim et al., 2015). Bowtie2 (v2.2.5) was applied to align the clean reads to the reference coding gene set. Then, the expression level of gene was calculated by RSEM (v1.2.12) (Li and Dewey, 2011). The heatmap was drawn by pheatmap (v1.0.8) according to the gene expression in different samples. Essentially, differential expression analysis was performed using the DESeq2(v1.4.5) (Love et al., 2014) with a Q value ≤ 0.05. To take insight to the change of phenotype, GO^1^ and KEGG^2^ enrichment analysis of annotated different expressed gene was performed by Phyper^3^ based on the Hypergeometric test. The significant levels of terms and pathways were corrected by Q value with a rigorous threshold (Q value ≤ 0.05) by Bonferroni.

### *In vitro* Fertilization (IVF)

The spermatozoa were suspended in the fertilization medium at a concentration of 1 × 10^6^ cells/ml and incubated at 38.5°C for one hour to capacitate. A total of 50 μL sperm sample was added to the fertilization droplets containing 30–35 matured oocytes, given to a final sperm with a concentration of 0.25 × 10^6^ cells/ml, and then incubated for 6 h. After fertilization, oocytes were washed for three times and cultured with 500 μL of porcine zygote medium in four-well dishes at 38.5°C with 5% CO_2_. Cleavage formation was evaluated on Day 2 after IVF and fertilization was determined to be successful.

### Parthenogenetic Activation

After being cultured for 44–48 h, oocytes were denuded of cumulus cells by pipetting in D-PBS containing 0.1% hyaluronidase, and those with intact first polar bodies were selected for electrical activation in a medium composed of 0.3 M mannitol, 0.05 mM CaCl_2_, 0.1 mM MgSO_4_, and 0.1% bovine serum albumin (BSA). Activation was induced with a twice DC pulse of 1.2 kV/cm for 40 μs. Oocytes were then incubated in PZM-3 medium with 5 μg/mL CB for three hours at 38.5°C under 5% CO_2_ in humidified air.

### Embryo Culture

Embryos were washed in D-PBS after treatment with CB, and then, according to the experimental design, embryos were cultured in PZM-3 at 38.5°C under 5% CO_2_ in humidified air. Cleavage rates and blastocyst rates were evaluated under a stereomicroscope at 2 and 6 days after activation or IVF, respectively.

### Statistical Analysis

At least three independently duplicated data were designated as mean percentages (mean ± SEM). Paired-samples *t*-test was performed for the differences between the two groups using the GraphPad Prism 6 statistical software. *P* < 0.05 was accepted to be significant.

## Results

### Different Doses of Cu Perturb the Porcine Oocyte Meiotic Maturation and Impair the Spindle/Chromosome Structure

**FIGURE 1:**
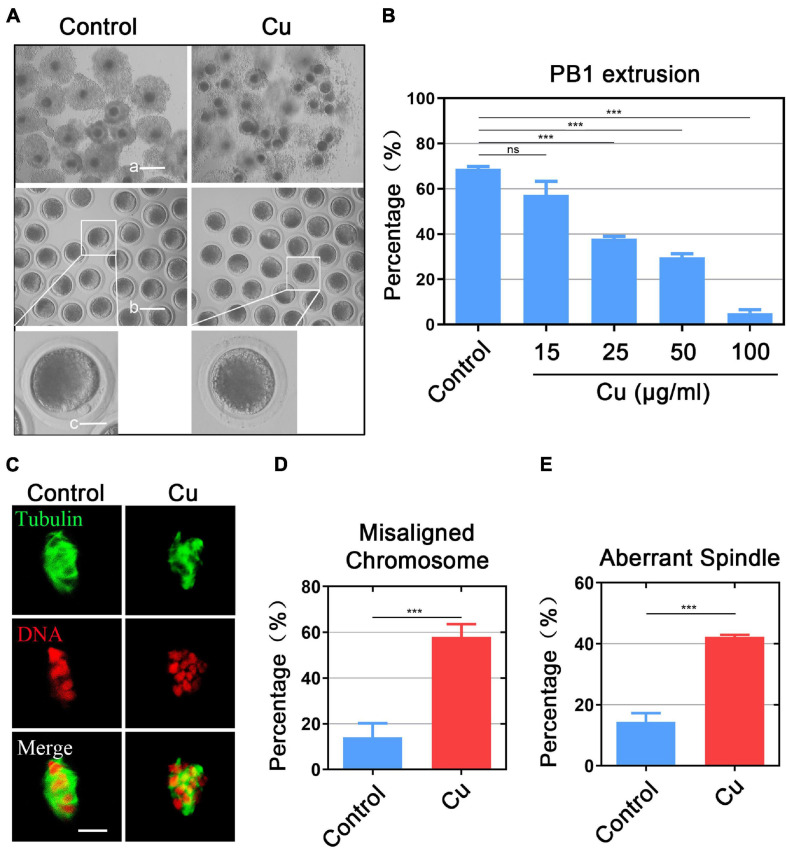
Different doses of Cu perturb the porcine oocyte meiotic maturation and impair the spindle/chromosome structure. **(A)** Representative images of oocyte meiotic progression in the control and Cu-exposed oocytes. Cumulus cell expansion of COCs and polar body extrusion of DOs (denuded oocytes) were imaged by the confocal microscope. Scale bar, 250 μm (a); 150 μm (b); 50 μm (c). **(B)** The rate of PBE was recorded in the control group and in different concentrations of the Cu-exposed groups (15, 25, 50, and 100 μg/mL) after culture for 44 h *in vitro*. **(C)** The spindles of oocytes were visualized by immunostaining with anti–α-tubulin-FITC antibody, and the chromosomes were visualized by counterstaining with propidium iodide (PI). Scale bar, 5 mm. **(D)** The rate of aberrant spindles was recorded in the control and Cu-exposed oocytes. **(E)** The rate of misaligned chromosomes was recorded in the control and Cu-exposed groups. Data in **(B,D,E)** were presented as the mean percentage of at least three independent experiments (mean ± SEM). *P < 0.05, **P < 0.01, ***P < 0.001, ns: no significance.

To confirm whether Cu exposure has effects on the porcine oocyte maturation, we chose different doses of Cu (including 15, 25, 50, and 100 μg/mL) which were added to the maturation medium during the *in vitro* maturation (IVM). As shown in Figures 1A,B, after culturing for 44 h *in vitro*, we found that most of the cumulus cells surrounding COCs in the control group were fully expanded, but those in the Cu-exposed group were not expanded or only partially expanded, and the frequency of the first polar body extrusion in the control group was higher than that in the Cu-exposed group (Figure 1A). Quantitative data displayed that the proportion of PBE was reduced in a dose-dependent manner after Cu exposure compared with the controls (68.8 ± 1.1%, *n* = 110, control vs. 57.3 ± 2.0%, *n* = 101, 15 μg/mL Cu, *P* = 0.133 vs. 37.9 ± 1.7%, *n* = 109, 25 μg/mL Cu, *P* < 0.0001 vs. 29.8 ± 1.6%, *n* = 119, 50 μg/mL Cu, *P* < 0.0001 vs. 5.1 ± 1.5%, *n* = 111, 100 μg/mL Cu, *P* < 0.0001; Figure 1B). We treated porcine COCs in the medium containing 25 μg/ml Cu for one hour, and then washed them out to observe the subsequent development in the fresh medium. The results showed that a short-term exposure to copper *in vitro* would not affect oocyte maturation (62.9 ± 1.6%, *n* = 99, control, vs. 58.3 ± 1.1%, *n* = 41, Cu, *P* = 0.18; Supplementary Figures 1A,B). We chose the concentration of 25 μg/mL Cu for further studies because a certain percentage of oocytes could be matured for the following studies at this concentration.

It is considered that the arrest of meiosis in oocytes is usually associated with the damage to cytoskeleton structures (Zhang et al., 2014, 2018), we examined the spindle structure and chromosome alignment in the Cu-exposed oocytes by staining. The results showed that a standard barrel-shape spindle structure and a well-aligned chromosome on the equatorial plate was exhibited in the control group (Figure 1C), while the Cu-exposed oocytes exhibited a higher frequency of aberrant spindle morphologies with misaligned chromosomes (misaligned chromosome: 14.0 ± 1.6%, *n* = 37 control, *P* < 0.001 vs. 58.1 ± 2.2%, *n* = 39, Cu; disorganized spindle: 14.3 ± 2.9%, *n* = 47, control, *P* < 0.001 vs. 42.3 ± 2.6%, *n* = 49, Cu; Figures 1D,E).

### Effects of Cu Exposure on the Distribution of Actin and Cortical Granules Dynamics in Porcine Oocytes

**FIGURE 2:**
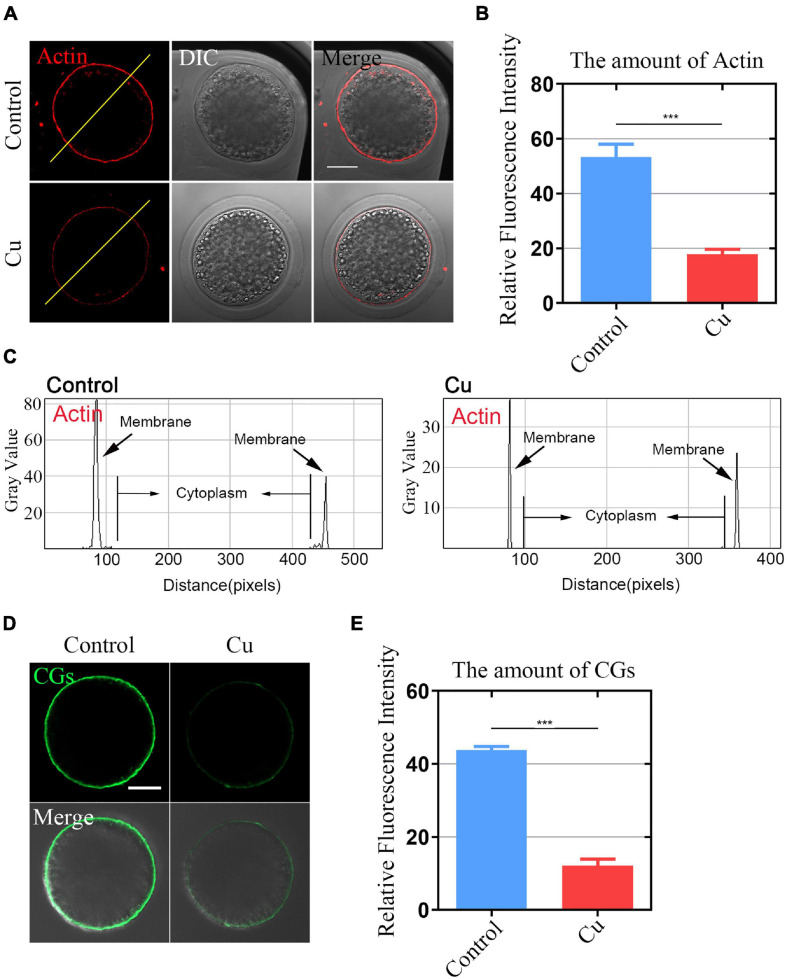
Effects of Cu exposure on the distribution of actin and cortical granules dynamics in porcine oocytes. **(A)** Representative images of actin filaments in the control and Cu-exposed oocytes. Scale bar, 30 mm. **(B)** The fluorescence intensity of actin signals was measured in the control and Cu-exposed oocytes. **(C)** Right graphs showed fluorescence intensity profiling of phalloidin in oocytes. Lines were drawn through the oocytes, and pixel intensities were quantified along the lines. **(D)** Representative images of cortical granule localization in the control and Cu-exposed oocytes. Scale bar, 20 mm. **(E)** The fluorescence intensity of cortical granules was measured in the control groups and the Cu-exposed oocytes. Data of **(B,E)** were presented as the mean percentage of at least three independent experiments (mean ± SEM). ***P < 0.001.

Actin assembly plays a key role in asymmetric spindle positioning and cortical polarization during the meiotic maturation of oocytes (Azoury et al., 2008; Pfender et al., 2011; Duan and Sun, 2019). In order to detect whether Cu exposure affects the actin dynamics of oocytes, phalloidin-TRITC was used to stain the F-actin to observe the polymerization of actin filaments. As shown in Figure 2A, in the control oocytes, actin filaments were uniformly concentrated on the plasma membrane with strong signals. However, in the Cu-exposed group, the assembly of actin filaments was impaired and the oocytes exhibited weak signals. The quantitative detection of relative fluorescence intensity on the oocyte’s membrane showed that the actin signals were drastically reduced in the Cu-exposed oocytes (50.9 ± 1.8, *n* = 39, control, *P* < 0.001 vs. 16.4 ± 1.2, *n* = 41, Cu; Figure 2B). In addition, the fluorescence plot analysis confirmed the results above by quantified along the lines drawn through the oocytes (Figure 2C).

The distribution of cortical granules (CGs) is one of the key indexes of oocytes cytoplasmic maturation (Ducibella et al., 1988). As shown in Figure 2D, in the Cu-exposed group, the signals of CGs exhibited a significant decrease. The quantitative detection of relative fluorescence intensity also confirmed that there was a markedly decline in the Cu-exposed oocytes compared to the controls (42.1 ± 2.3, *n* = 29, control, *P* < 0.001 vs. 10.2 ± 1.9, *n* = 31, Cu; Figure 2E).

### Effects of Cu on the Fertilization Ability and Early Embryo Development of Porcine Oocytes

**FIGURE 3:**
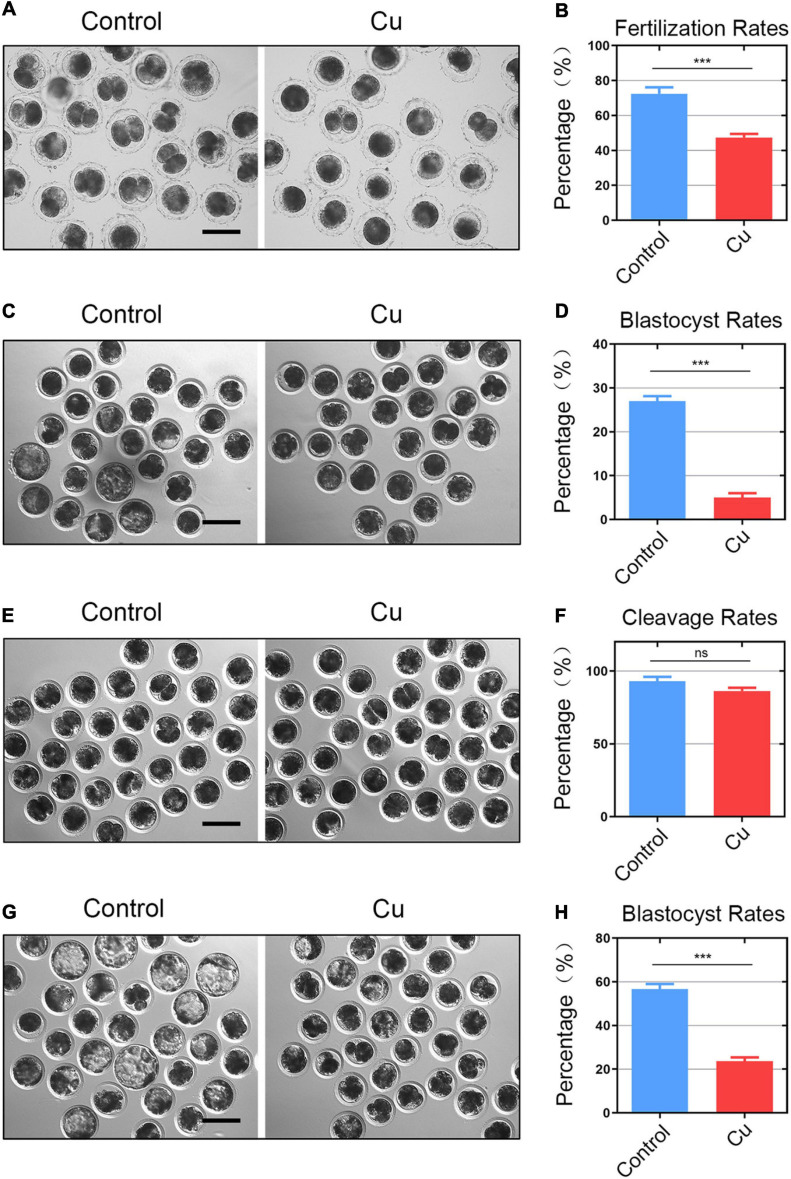
Effects of Cu on the fertilization ability and early embryo development of porcine oocytes. **(A)** Representative images of the fertilized eggs in the control and Cu-exposed groups. Scale bar, 120 μm **(B)** *In vitro* fertilization rate was recorded in the control groups and the Cu-exposed oocytes. **(C)** Representative images of the blastocyst rate in the control and Cu-exposed groups after IVF. Scale bar, 100 μm **(D)** blastocyst rate was recorded in the control groups and the Cu-exposed oocytes after IVF. **(E)** Representative images of the parthenogenetic activation rate in the control and Cu-exposed groups. Scale bar, 100 μm. **(F)** Parthenogenetic activation rate was recorded in the control groups and the Cu-exposed oocytes. **(G)** Representative images of the blastocyst rate in the control and Cu-exposed groups after parthenogenetic activation. Scale bar, 100 μm **(H)** blastocyst rate was recorded in the control groups and the Cu-exposed oocytes after parthenogenetic activation. Data of **(B,D,F,H)** were presented as the mean percentage of at least three independent experiments (mean ± SEM). ns: no significance; ***P < 0.001.

Decreased porcine oocytes quality caused by Cu exposure can affect the fertilization ability and embryonic development. We further carried out an *in vitro* fertilization to confirm it. The result was as expected (Figure 3A). The number of two-cell embryos in the Cu-exposed group was significantly decreased compared to the control group (72.5 ± 1.6%, *n* = 40, control vs. 47.4 ± 2.0%, *n* = 38, Cu, *P* < 0.0001; Figure 3B). The blastocyst rate after IVF in the Cu-exposed group was significantly decreased compared to the control group (26.5 ± 1.5%, *n* = 81, control vs. 6.3 ± 2.1%, *n* = 88, Cu, *P* < 0.0001; Figures 3C,D). Parthenogenetic activation experiment proved that a high concentration of copper did not affect the parthenogenetic activation rate of oocytes (93.0 ± 2.6%, *n* = 180, control vs. 86.3 ± 2.7%, *n* = 211, Cu, *P* < 0.005; Figures 3E,F). However, it had a significant effect on the embryonic development (Figure 3G). The blastocyst rate after parthenogenetic activation in the Cu-exposed group was significantly decreased compared to the control group (56.7 ± 2.1%, *n* = 101, control vs. 23.7 ± 2.9%, *n* = 117, Cu, *P* < 0.0001; Figure 3H). The results of the parthenogenetic activation experiments by means of electrical activation showed that a high concentration of copper did not affect the parthenogenetic activation rate of oocytes. However, a high concentration of copper affected the fertilization ability and blastocyst rate after the parthenogenetic activation or IVF of porcine oocytes.

### Transcriptome Analysis Identifies the Target Effectors of Cu in Porcine Oocytes

**FIGURE 4:**
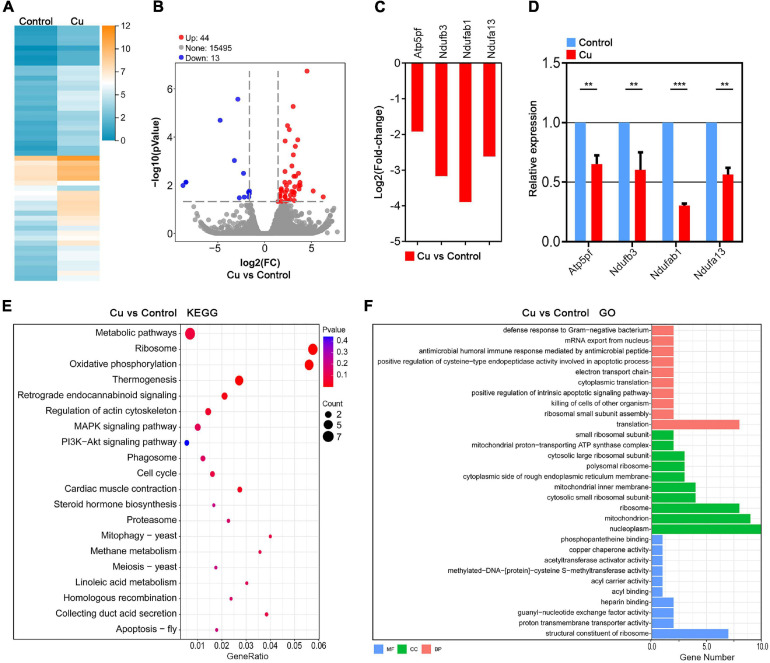
Transcriptome analysis identifies target effectors of Cu in porcine oocytes. **(A)** Heatmap illustration showing the gene expression of the control and Cu-exposed groups. **(B)** Volcano plot showing the differentially expressed genes (DEGs; upregulated, red; downregulated, blue) in the control compared with Cu-exposed oocytes. **(C)** RNA-seq results of the selected genes in the Cu-exposed oocytes compared with the control ones (red). **(D)** Validation of the RNA-seq data by quantitative RT-PCR in the control oocytes (blue) and the Cu-exposed oocytes (red). Data were presented as the mean percentage of at least three independent experiments (mean ± SEM). **P < 0.01, ***P < 0.001. **(E)** KEGG enrichment analysis of DEGs in the Cu-exposed oocytes compared with the control ones. **(F)** GO enrichment analysis of DEGs in the Cu-exposed oocytes compared with the control ones. Orange represents biological process; blue represents molecular function; and green represents cellular component.

In order to further investigate the underlying mechanisms of the effects of Cu supplementation on the porcine oocytes, the single-cell transcriptome analysis was performed to identify the target effector of Cu. Heatmap and volcano plot data showed that the transcriptome profile of the Cu-exposed oocytes was different from that of the control oocytes, exhibiting that 44 differentially expressed genes (DEGs) were upregulated and 13 DEGs were downregulated in the Cu-exposed oocytes (Figures 4A,B). Four randomly selected genes in the downregulated group were verified using Log2(Fold-change) analysis and quantitative real-time PCR. As shown in Figures 4C,D, the expression of four genes (*Atp5pf*, *Ndufb3*, *Ndufab1*, *Ndufa13*) were significantly downregulated in the Cu-exposed oocytes compared to the controls.

Kyoto Encyclopedia of Genes and Genomes (KEGG) analysis of DEGs revealed the abnormal expression of genes enriched in the oxidative phosphorylation pathway and regulation of actin cytoskeleton pathway in the Cu-exposed oocytes compared with the control groups (Figure 4E). In addition, Gene Ontology (GO) analysis showed that DEGs were associated with the mitochondrial function including the mitochondrial proton-transporting ATP synthase complex, mitochondrial inner membrane, and mitochondrion (Figure 4F). In summary, the Cu-exposed oocytes that exhibited an abnormality are highly related to oxidative stress and mitochondrial function, which prompts us to focus on mitochondrial function and oxidative stress as the target effectors of copper in porcine oocytes.

### Effects of Cu Exposure on the Distribution and Function of Mitochondria in Porcine Oocytes

**FIGURE 5:**
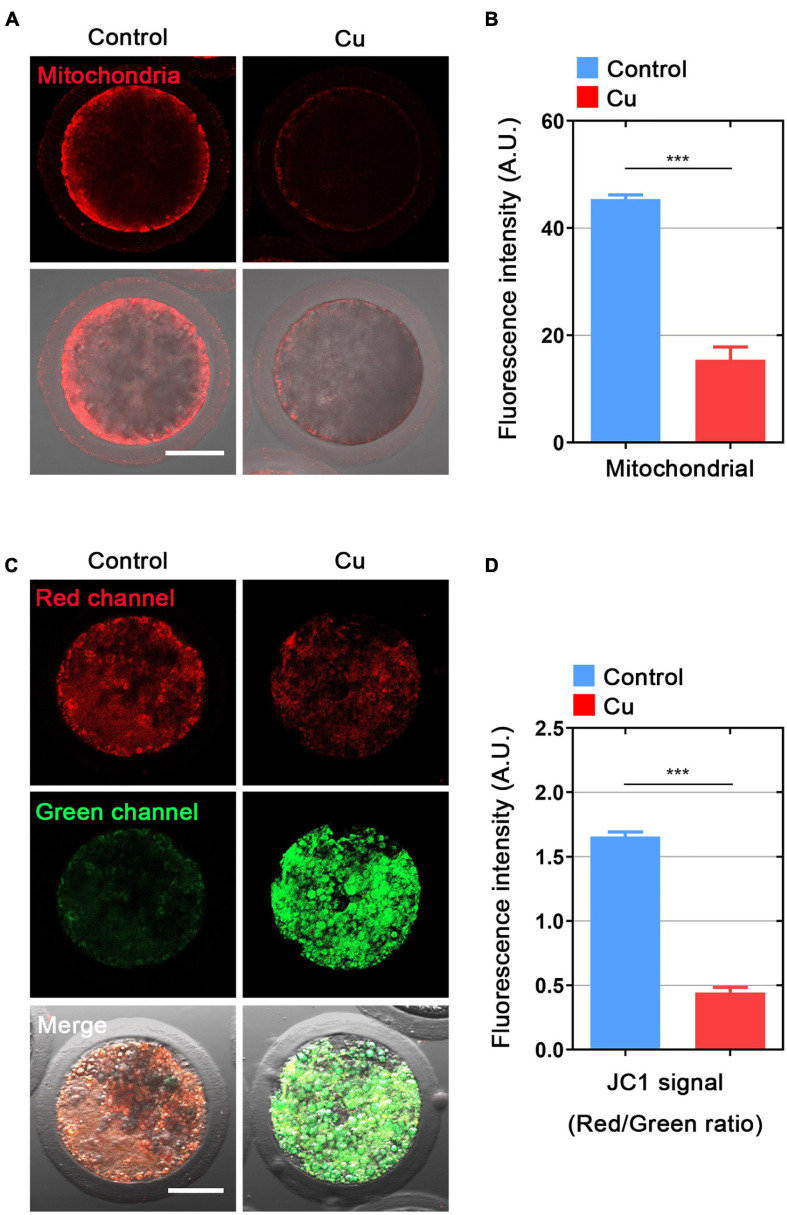
Effects of Cu exposure on the distribution and function of mitochondria in porcine oocytes. **(A)** Representative images of mitochondria in the control and Cu-exposed oocytes. Scale bar, 50 μm. **(B)** The immunofluorescence intensity of mitochondrion signals was recorded in the control and Cu-exposed oocytes. **(C)** Mitochondrial membrane potential (ΔΨm) was detected by JC-1 in the control and Cu-exposed oocytes (Green, low ΔΨm; Red, high ΔΨm). **(D)** The ratio of red and green fluorescence intensity was recorded in the control and Cu-exposed oocytes. Data of **(B)** and **(D)** were expressed as the mean percentage of at least three independent experiments (mean ± SEM). ***P < 0.001.

The distribution of mitochondria is another key index of the cytoplasmic maturation of oocytes (Sun et al., 2001). In the porcine oocytes in the control group, most of mitochondria accumulated around lipid droplets in the subcortical region, but the Cu-exposed oocytes mitochondria lost its specific localization (Figure 5A). The quantitative detection of relative fluorescence intensity showed that the mitochondria signals were drastically reduced in the Cu-exposed oocytes compared to the controls (45.5 ± 2.0, *n* = 29, control vs. 16.9 ± 2.2, *n* = 28, Cu, *P* < 0.0001; Figure 5B). In addition, the mitochondria membrane potential (ΔΨm) was detected by JC-1 staining. High membrane potential mitochondria showed a red fluorescence, while low membrane potential mitochondria showed a green fluorescence (Figure 5C). The quantitative detection of the relative fluorescence intensity showed that the ratio of red and green fluorescence was significantly lower in the Cu-exposed oocytes than that in the controls (1.64 ± 1.2, *n* = 26, control vs. 0.45 ± 1.3, *n* = 21, Cu, *P* < 0.0001; Figure 5D). Altogether, this part of the results further verified the conclusion of the single cell transcriptome data analysis: a high concentration of copper treatment caused damage to the distribution and function of mitochondria in oocytes.

### Effects of Cu Exposure on the ROS Level, DNA Damage, and Early Apoptosis in Porcine Oocytes

**FIGURE 6:**
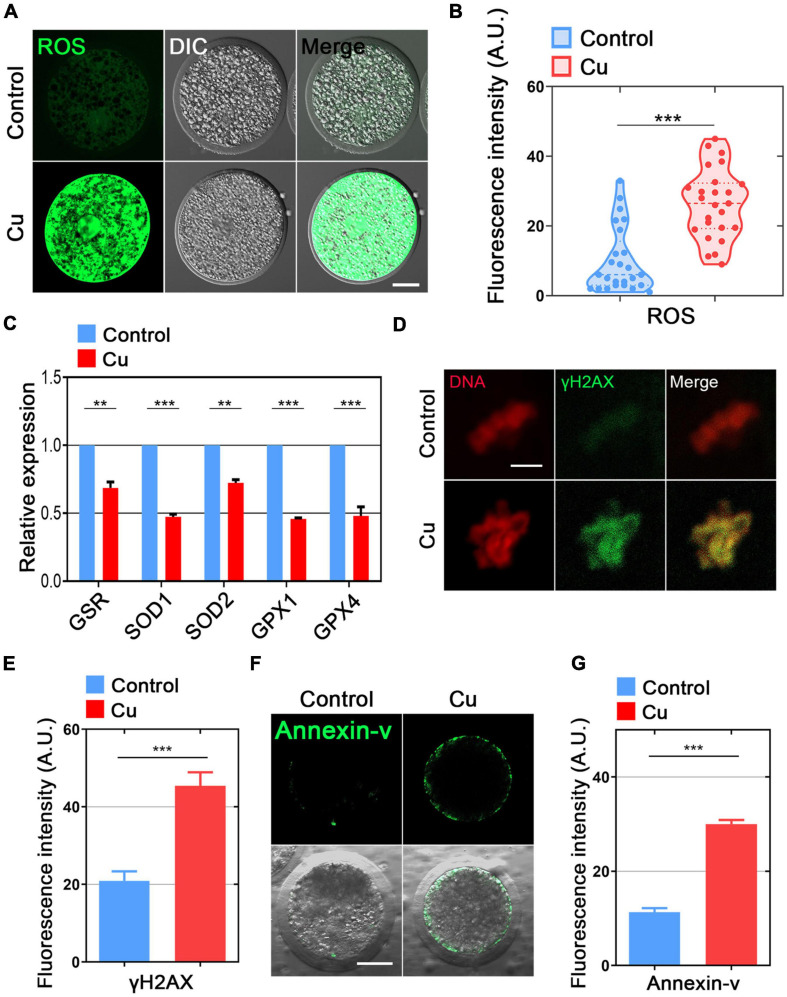
Effects of Cu exposure on the ROS level, DNA damage, and early apoptosis in porcine oocytes. **(A)** Representative images of ROS signals in the control and Cu-exposed oocytes. Scale bar, 30 μm. **(B)** The fluorescence intensity of ROS was quantified in the control and Cu-exposed oocytes. **(C)** The expression of genes related to the anti-oxidation pathway was examined in the control and Cu-exposed oocytes. **(D)** The representative images of DNA damagm. **(B)** The fluorescence intensity of ROS was quantified in the control and Cu-exposed oocytes. **(C)** The expression of genes related to the anti-oxidation pathway was examined in the control and Cu-exposed oocytes. **(D)** The representative images of DNA damage in the control and Cu-exposed oocytes. Scale bar, 5 μm. **(E)** The fluorescence intensity of γH2A.X signals was quantified in the control and Cu-exposed oocytes. **(F)** The representative images of apoptotic oocytes were shown in the control and Cu-exposed oocytes. Scale bar, 30 in the control and Cu-exposed oocytes. Data of **(B,C,E,G)** were expressed as the mean percentage of at least three independent experiments (mean ± SEM). **P < 0.01, ***P < 0.001.

Mitochondrial dysfunction is known to cause ROS production and oxidative stress; therefore, we used dichlorofluorescein (DCFH) staining to compare the ROS levels in porcine oocytes from the control and the Cu-exposed groups (Figure 6A). The results showed that the fluorescence intensity of ROS signal in the Cu-exposed oocytes was significantly stronger than that of the control group (9.8 ± 1.8, *n* = 25, control vs. 26.4 ± 1.9, *n* = 25, Cu, *P* < 0.0001; Figure 6B). In addition, several genes involved in the antioxidant pathways were verified using quantitative real-time PCR. As shown in Figure 6C, the expression of five genes (*GSR*, *SOD1*, *SOD2*, *GPX1*, *GPX4*) was significantly downregulated in the Cu-exposed oocytes compared to the controls.

High levels of oxidative stress often cause DNA damage and early apoptosis (Ratan et al., 1994; Ozben, 2007). Next, we chose γ-H2A.X staining and Annexin-V staining to verify the DNA damage and early apoptosis in the Cu-exposed oocytes and the controls. The immunofluorescence results showed that the γ-H2A.X signals and Annexin-V signals was remarkably higher in the Cu-exposed oocytes than that of the control groups (Figures 6D,F), and the fluorescence intensity analysis also confirmed the result above (20.6 ± 1.2, *n* = 22, control vs. 46.1 ± 2.4, *n* = 19, Cu, *P* < 0.0001; Figure 6E; 11.9 ± 1.9, *n* = 19, control vs. 30.9 ± 2.1, *n* = 18, Cu, *P* < 0.0001; Figure 6G).

## Discussion

It has been previously found that Cu promoted the growth rate of pigs (Cromwell et al., 1989), and a high Cu diet is widely used in pig production. However, excessive dietary Cu can seriously affect the growth of livestock and lead to serious environmental problems through excretion in feces (Sutton and Richert, 2004). At present, there has been limited research of Cu actions in reproduction. Some studies showed that trace amounts of Cu (0, 2, 4, and 6 μg/mL) during the *in vitro* maturation of bovine oocytes can improve the content of glutathione and DNA integrity in cumulus cells, and reported that the optimal development of embryos to the blastocyst stage depends on the addition of Cu during the *in vitro* maturation (Picco et al., 2012). In addition, previous reports showed that dietary Cu supplementation enhanced the *in vitro* fertilization ability of frozen semen from goats. The zona pellucida binding and embryo cleavage rates for the *in vitro* fertilization with goat sperm were significantly higher in the Cu-treated groups than in the control groups, and there was a dose-dependent response (Hackbart et al., 2010). Choi et al. (2021) treated porcine oocytes with 2.8 μg/ml copper and found that Cu supplementation at appropriate concentrations in the IVM medium improved porcine oocyte maturation and the subsequent embryonic potential. They showed that the mRNA levels of Has-2, a cumulus cell expansion-related gene, were higher in all the Cu-treated groups than that in the control group. In the 0.7 μg/ml Cu group, the mRNA expression levels of PCNA, ZAR1, and NPM2, which are related to developmental competence, were significantly higher than those in the control group. Moreover, increased levels of SOD1 transcript, correlated with the antioxidative response, were observed in the 0.7 and 1.4 μg/ml Cu groups. However, the effects of Cu, in particular excessive Cu levels, on the reproductive performance of boars and sows have not been studied.

Two key indicators of normal maturation of porcine oocyte are polar body extrusion and cumulus cells expansion. We have shown that increasing the Cu sulfate concentration (15, 25, 50, and 100 μg/mL) during the *in vitro* culture of porcine oocytes led to the abnormal expansion of cumulus cells and a decreased oocyte maturation rate. Since the breakdown of cytoskeletal assembly usually results in cell division defects in both meiotic and mitotic cells (Azoury et al., 2008; Heng and Koh, 2010), we investigated the negative effects of Cu exposure on the oocyte meiotic progression by studying cytoskeleton dynamics. Our data showed that Cu exposure disrupted spindle organization by impairing the microtubules stability, and with the chromosomal misalignment. In addition, Cu exposure destroyed the integrity of actin and cortical granules, both essential components for cytoskeletal and cytoplasmic maturation. Thus, these findings demonstrated that a disrupted oocyte maturation induced by Cu exposure was attributable to a deficiency in the cytoskeleton dynamics. Mature porcine oocytes treated with Cu exhibited a significantly decreased fertilization rate compared with the controls, showing that a high concentration of Cu reduced the quality and the fertilization ability of oocytes. Fertilization is a complicated event that requires energy supply. High concentrations of copper impair the function of the mitochondria, which provide energy to cells and maintain a redox balance. The accumulation of ROS is one of the reasons for the abnormal transport of cytoskeleton and cortical granules (Miao et al., 2018a, b). Cortical granules are involved in the cortical reaction during the process of fertilization, and inhibit the occurrence of polyspermy. Further studies of embryo development showed that 25 μg/mL of copper treatment also significantly reduced embryo development ability after IVF. We speculate that the decrease of oocyte quality is the direct cause of an abnormal embryo development. To validate this, we further verified that the blastocyst rate of oocytes treated with a high concentration of copper was significantly lower than that of the control group by parthenogenetic activation. However parthenogenetic activation did not affect the activation rate of porcine oocytes, indicating that a high concentration of copper did not affect the signal pathway or calcium ion channels during the electrical activation of oocytes (Cheng et al., 2007; Saadeldin et al., 2018).

The data of single-cell transcriptome analysis showed that genes related to cytoskeleton, such as ITGB5, GNG12, and PIP4K2B, were affected by copper treatment. Normal oocyte cytoplasmic maturation are indicated by the migration of cortical granules from the center region to the subcortical region following their synthesis from the Golgi apparatus (Ahmed et al., 2017), and this translocation is mediated by the actin filaments. Therefore, copper treatment might affect the cytoskeleton dynamics and cytoplasmic transport, resulting in the defective distribution of cortical granules to the subcortical area of oocytes. In addition, the single-cell transcriptome data further showed that genes in the oxidative phosphorylation pathway and mitochondrial organization-related processes were significantly downregulated in the Cu-exposed oocytes, suggesting that the mitochondria were the downstream effector of Cu actions. In agreement with the role of mitochondria, we found that Cu exposure impaired the normal mitochondrial distribution pattern, as well as mitochondrial function, including the membrane potential and ATP production capacity (Grootegoed et al., 1984; Cai et al., 2015; Zhang et al., 2019; Miao et al., 2020). Mitochondria play a primary role in oocytes and are an important source of the generation of ATP for cell development (Dumollard et al., 2007; Niu et al., 2019), and the mitochondrial distribution pattern is considered a critical indicator for evaluating oocyte cytoplasmic maturation. Our findings indicated that Cu exposure impaired cytoplasmic maturation of oocytes by disrupting the mitochondrial distribution and function. As a result, increased ROS levels induced the DNA damage and apoptosis accumulation in the Cu-exposed oocytes.

## Conclusion

All in all, we provide a substantial record of evidence that Cu exposure results in mitochondrial dysfunction and redox perturbation in porcine oocytes, thereby generating ROS accumulation-induced apoptosis, which is a main cause for a deteriorated oocyte quality.

## Data Availability Statement

The data presented in the study are deposited in the (https://www.ncbi.nlm.nih.gov/) repository, accession number (GSE168867).

## Ethics Statement

The animal study was reviewed and approved by the Animal Care and Use Committee of Nanjing Agricultural University.

## Author Contributions

QG and YM designed the research. JC, ZC, YQ, XZ, FC, and HW performed the experiments. QG, YM, and BX analyzed the data. JC, QG, and YM wrote the manuscript. All authors contributed to the article and approved the submitted version.

## Conflict of Interest

The authors declare that the research was conducted in the absence of any commercial or financial relationships that could be construed as a potential conflict of interest.

## Footnotes

1 http://www.geneontology.org/

2 https://www.kegg.jp/

3 https://en.wikipedia.org/wiki/Hypergeometric_distribution
