# Supplementary material for: Exposure to Copper Compromises the Maturational Competency of Porcine Oocytes by Impairing Mitochondrial Function
Source: Front Cell Dev Biol. 2021 Jun 4;9:678665. doi: 10.3389/fcell.2021.678665 (PMC8212058; doi:10.3389/fcell.2021.678665)
Supplement: Supplementary file 1 [file Data_Sheet_1.docx]

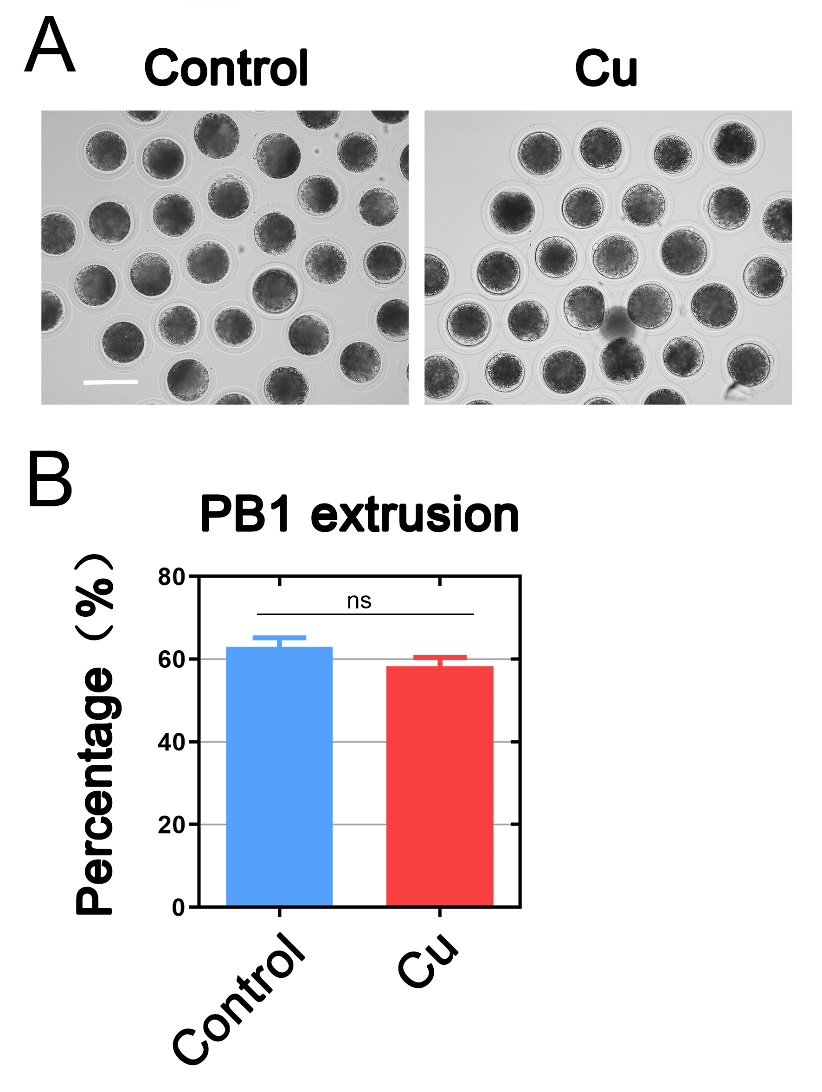


**Figure S1.** Effect of short incubation of Cu on the porcine oocyte meiotic maturation. (A) Representative images of oocyte meiotic progression in control and short-term Cu exposure oocytes. Polar body extrusion of DOs (denuded oocytes) were imaged by the confocal microscope. Scale bar, 150 μm. (B) The rate of PBE was recorded in control and short-term Cu exposure groups (25 μg/ml Cu for 1 h) after culture for 44 h in vitro. Data in B was presented as the mean percentage of at least three independent experiments (mean ± SEM). ns: no significance.
